# Quantum-dot supercrystals for future nanophotonics

**DOI:** 10.1038/srep01727

**Published:** 2013-04-25

**Authors:** Anvar S. Baimuratov, Ivan D. Rukhlenko, Vadim K. Turkov, Alexander V. Baranov, Anatoly V. Fedorov

**Affiliations:** 1 National Research University of Information Technologies, Mechanics and Optics, 49 Kronverksky Avenue, Saint Petersburg 197101, Russia; 2 Advanced Computing and Simulation Laboratory (AχL), Department of Electrical and Computer Systems Engineering, Monash University, Clayton, VIC 3800, Australia

## Abstract

The study of supercrystals made of periodically arranged semiconductor quantum dots is essential for the advancement of emerging nanophotonics technologies. By combining the strong spatial confinement of elementary excitations inside quantum dots and exceptional design flexibility, quantum-dot supercrystals provide broad opportunities for engineering desired optical responses and developing superior light manipulation techniques on the nanoscale. Here we suggest tailoring the energy spectrum and wave functions of the supercrystals' collective excitations through the variation of different structural and material parameters. In particular, by calculating the excitonic spectra of quantum dots assembled in two-dimensional Bravais lattices we demonstrate a wide variety of spectrum transformation scenarios upon alterations in the quantum dot arrangement. This feature offers unprecedented control over the supercrystal's electromagnetic properties and enables the development of new nanophotonics materials and devices.

The creation of artificial materials with prescribed physical properties that cannot be found in nature is one of the greatest challenges of the contemporary science and engineering. Recently the field of photonics has been significantly enriched by the successful design and fabrication of photonic crystals^1^,^2^,^3^ and optical metamaterials^4^,^5^,^6^. These man-made structures acquire their physical properties not so much due to their composition as owing to the periodic arrangement or design of the subwavelength constituent parts. This has enabled scientists and engineers to change their way of thinking about light–matter interaction, and revolutionized the design paradigm of photonics devices^7^,^8^,^9^.

Modern nanofabrication technology allows artificial materials to be created with hundreds and even thousands of nanoscale building blocks of extreme intricacy and fine detail. One of the most promising types of such building blocks—semiconductor quantum dots—is also referred to as ‘artificial atoms’ due to the discrete energy spectra of their elementary excitations such as electrons (holes), excitons, phonons, and polaritons^10^,^11^,^12^. Adamant interest in quantum dots over the past few decades is explained by their unique physical properties^13^,^14^,^15^,^16^ and the possibility of modifying them by varying the quantum dot's shape and dimensions. The linear and nonlinear responses of their electronic and vibrational subsystems^17^,^18^,^19^,^20^,^21^—as well as the interaction of quantum dots with each other and external electromagnetic fields^22^,^23^,^24^,^25^—are all drastically dependent on the size and geometry of the quantum dots. These dependencies find applications in various electronic and optoelectronic devices, including quantum-dot lasers^26^,^27^,^28^, q-bits for quantum computing and information processing^29^,^30^,^31^, single-photon sources^32^,^33^,^34^, solar cells^35^,^36^,^37^, and photodetectors^38^,^39^,^40^. Although certain photonic functions can be conveniently realized with a single quantum dot, the ensembles of coupled quantum dots arranged periodically^41^,^42^,^43^,^44^,^45^,^46^ are much more versatile from the application viewpoint. Just as optical metamaterials change their response with modifications to meta-atoms, such *quantum-dot supercrystals*^47^,^48^,^49^,^50^ can be engineered to exhibit properties beneficial for nanophotonics applications by rearranging quantum dots and varying their parameters.

It is of significance that the quantum-dot supercrystals can be fabricated using many versatile techniques, including the Langmuir–Blodgett fabrication^51^,^52^, molecular beam epitaxy (MBE)^53^, nonlithographic formation by anodic membrane template^54^, DNA-assisted formation^55^, self-assembly of colloidal nanocrystals^56^,^57^,^58^,^59^,^60^,^61^, and the method of ion-beam-assisted self-assembly^62^. For example, in the last case the irradiation of an amorphous multilayer with an ion beam allows one to readily create ordered quantum-dot arrays and control structural properties and arrangement of the quantum dots by tuning the angle between the ion beam and the multilayer surface.

This paper aims to instigate extensive research of artificial materials enabled by quantum-dot supercrystals. Using the theory of molecular crystals, we demonstrate almost unlimited opportunities for engineering the quantum states of the supercrystals and—as a consequence—their physical properties. These opportunities arise due to the multiple degrees of freedom associated with the possibility to preset the properties of individual quantum dots and their mutual arrangement. In particular, we calculate the energy spectra corresponding to four simple two-dimensional Bravais lattices and a complex lattice with two quantum dots in a basis, and thoroughly analyze the transformation of these spectra with varying structural parameters. Our results suggests quantum-dot supercrystals as unique material base for the new-generation nanophotonics devices.

## Results

### Theoretical formulation

One of the most powerful tools for studying collective excitations in the ensembles of periodically arranged semiconductor quantum dots is the method of modeling exciton states in molecular crystals^63^,^64^. The essence of this method, as applied to a quantum-dot supercrystal, is illustrated by the following example.

**Figure 1:**
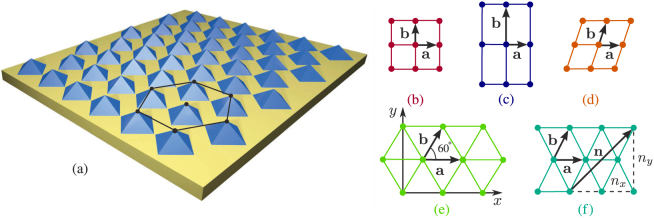
(a) Schematic of two-dimensional supercrystal made of pyramidal quantum dots arranged in a hexagonal lattice. A simple two-dimensional supercrystal have either a (b) square, (c) rectangular, (d) oblique, (e) hexagonal, or (f) centered rectangular Bravais lattice, each of which is characterized by the translation vectors **a** and **b**.

Suppose that *N* quantum dots form a two-dimensional simple lattice (supercrystal) characterized by the primitive vectors **a** and **b** (see Fig. 1), so that each quantum dot may be denoted by its position vector **n** = *n*_1_**a** + *n*_2_**b**, with *n*_1_ and *n*_2_ being integers. If 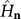 is the Hamiltonian of an isolated quantum dot **n**, whose interaction with dot **m** is described by operator 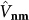, then the Hamiltonian of the supercrystal's collective excitations can be written in the form


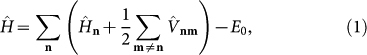
where the energy *E*_0_ of the supercrystal's ground state includes the energies of the quantum dots' carriers in their ground states and the energy of interaction between these carriers.

It is reasonable to assume that the wave functions of the low-energy electronic states of the neighboring quantum dots do not overlap significantly, and neglect the exchange interaction^63^,^64^. Then the ground-state wave function Ψ_0_ of the supercrystal is the product of the wave functions 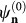 describing the ground states of individual nanocrystals, 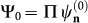. In turn, each wave function 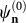 is a single Slater determinant,


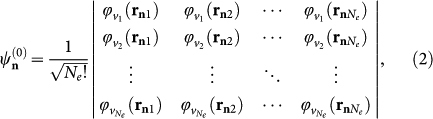
which is the antisymmetrized—with respect to all possible exchanges of *N_e_* electrons—product of one-electron wavefunctions 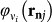, where the subscript ν_*i*_ denotes a set of quantum numbers and **r_n_**_*j*_ is the position of the *j*th electron. It should be noted that the assumption of negligible exchange interaction strictly holds for self-organized colloidal nanocrystals providing high potential barriers for their electrons and holes^65^,^66^, but may be violated for supercrystals fabricated using MBE^53^ or ion-beam-assisted self-assembly^62^.

The function 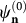 describes a quantum dot with fully occupied valence band *v* and an empty conduction band *c*, and is the solution of the Schrödinger equation 
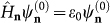
 corresponding to the lowest energy *ε*_0_ of the quantum dot's electronic subsystem. With these notations, we obtain


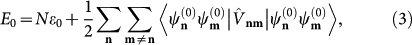
where the integration in the matrix element is over the coordinates of electrons in quantum dots **n** and **m**.

Let an electron in a quantum dot **n** be excited from its *μ_v_*-fold degenerate valence-band state 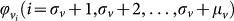 to the *μ_c_*-fold degenerate conduction-band state 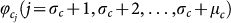. Such an ‘excited’ quantum dot is described by the wave function


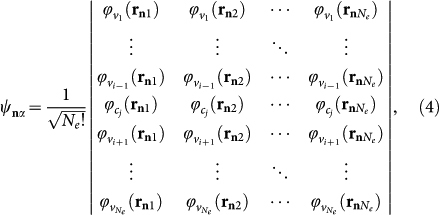
which depends on double subscript α = {*i, j*} (α = 1, 2, …, *μ_c_* × *μ_v_*) and is related to the quantum dot's energy *ε via* the equation 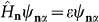. In the absence of an interdot interaction, the eigenstate of the supercrystal with an excited quantum dot **n** is a *μ_c_* × *μ_v_* × *N*-fold degenerate, has energy *ε* + (*N* − 1)*ε*_0_, and is described by the wave function


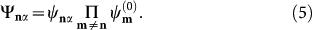
The energy spectrum 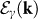 and wave functions Φ_γ_(**k**) of the real supercrystal can be found by diagonalizing the operator of the interdot interaction using the linear combinations of functions Ψ_**n**α_. Since these combinations must be eigenfunctions of the translation operator, they are of the form


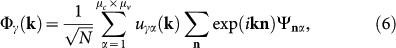
where **k** is the wave vector of the supercrystal's excitation and *u*_γα_(**k**) are the elements of a unitary matrix.

By requiring Φ*_γ_*(**k**) to be the eigenfunctions of the Hamiltonian in Eq. (1) and using the Heitler–London approximation^63^,^64^, we arrive at the following set of linear equations:


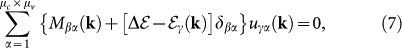
where


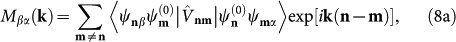

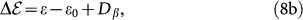
and


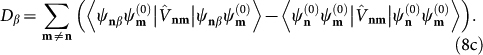
Since *M_βα_*(**k**) is the Hermitian matrix, equating the determinant of Eq. (7) to zero may give maximum *μ_c_* × *μ_v_* real roots 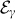 corresponding to *μ_c_* × *μ_v_* exciton bands of the supercrystal. The degeneracy of these bands (if any) depends on the symmetry of the interaction coupling the quantum dots, as well as on the dimension of the irreducieable representations of the supercrystal's symmetry group^64^.

Once the spectrum and wave functions of the supercrystal's excitons are known, one can calculate any physical property of the supercrystal that is dependent on the state of its electronic subsystem, e.g. linear permittivity *ε_L_* or third-order nonlinear susceptibility *χ*^(3)^ 67.

### Exciton bands of two-dimensional supercrystals

The above theory may be applied to study the excitons of quantum-dot supercrystals and illustrate the possibility of engineering their dispersion by varying the arrangement and properties of the quantum dots.

To a first approximation, the coupling of electrically neutral quantum dots located at points **n** and **m** of a two-dimensional simple lattice is caused by the dipole–dipole interaction of their electrons. If the positions of the electrons in the reference frame of the supercrystal are given by the radius-vectors **r_n_** and **r_m_**, which are measured from the quantum dots' centers, then the operator of the dipole–dipole interaction can be written as


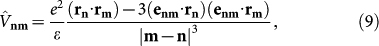
where *e* is the charge of a free electron, **e_nm_** = (**m** − **n**)/|**m** − **n**|, and ε is the effective permittivity, which takes into account the screening of the interaction potential by the quantum dots, the dielectric substrate, and the host medium. In the case of spherical quantum dots of high-frequency permittivity ε_QD_ embedded in the host of high-frequency permittivity ε_*h*_, we have ε = (ε_QD_ + 2ε_*h*_)^2^/(9ε_*h*_)^68^.

To calculate the matrix elements entering Eq. (7), one needs first to express the electron's radius-vector **r_n_** ≡ (*x*, *y*, *z*) through the radius-vector **R_n_** ≡ (*X*, *Y*, *Z*) of the same electron in the crystallographic system. This can done by rotating the crystallographic system of dot **n** by a certain angle *ψ***_n_** around a unit vector **u**(*ϑ***_n_**, *φ***_n_**), whose direction is determined by the polar angle *ϑ***_n_** and azimuth *φ***_n_**^69^. Such a coordinate transformation is described by the rotation tensor 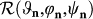 *via* the relation 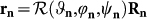.

Consider the lowest-energy dipole-allowed states of the quantum dots. The optical transitions involving these states exhibit the minimal dephasing rates, which facilitates the formation of the coherent exciton states of the supercrystal. We restrict our consideration to the three-band model^70^ of the valence band, in which case Eq. (7) can be solved analytically. Suppose that the wave function |*S*〉 of the conduction band is fully symmetric at the Brillouin zone center, while the valence band is triply degenerate and described by the wave functions |*X*〉, |*Y*〉, and |*Z*〉 with the symmetries of the respective coordinates of the crystallographic system. If we ignore the spin of the electron, this situation corresponds to *μ_v_* = 3 and *μ_c_* = 1. In the case where the crystallographic axes of all quantum dots are oriented in space identically, the energies of the exciton bands do not depend on the angles *ϑ*, *φ*, and *ψ*. According to Eq. (7), these energies are of the form


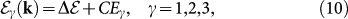
where *C* = 2*e*^2^〈*S|Z|Z*〉^2^/(*εa*^3^), 〈*S|Z|Z*〉 = 〈*S|Y|Y*〉 = 〈*S|X|X*〉 is the matrix element of the electron's coordinate, and *E*_γ_ is the dimensionless function representing the wave vector dependence of the exciton's energy. In the following, for brevity, we shall be refereing to *E*_γ_ as the exciton band energy.

Some algebra shows that the exciton energies for the four Bravais lattices with mirror reflection planes *x* = 0 and *y* = 0 are given by the expressions


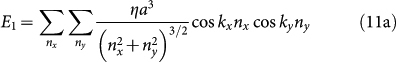
and


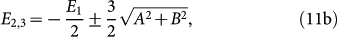
where 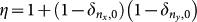, δ*_ij_* is the Kronecker delta,


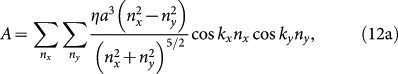
and


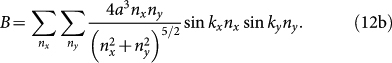
Here the summations are evaluated over the coordinates of all quantum dots in the first quadrant of the two-dimensional Cartesian system, including the dots lying on the coordinate axis, but excluding the dot located at the origin.

We now employ the obtained expressions to demonstrate the possibility of engineering exciton bands in quantum-dot supercrystals of different symmetries.

### Supercrystals with square and rectangular lattices

**Figure 2:**
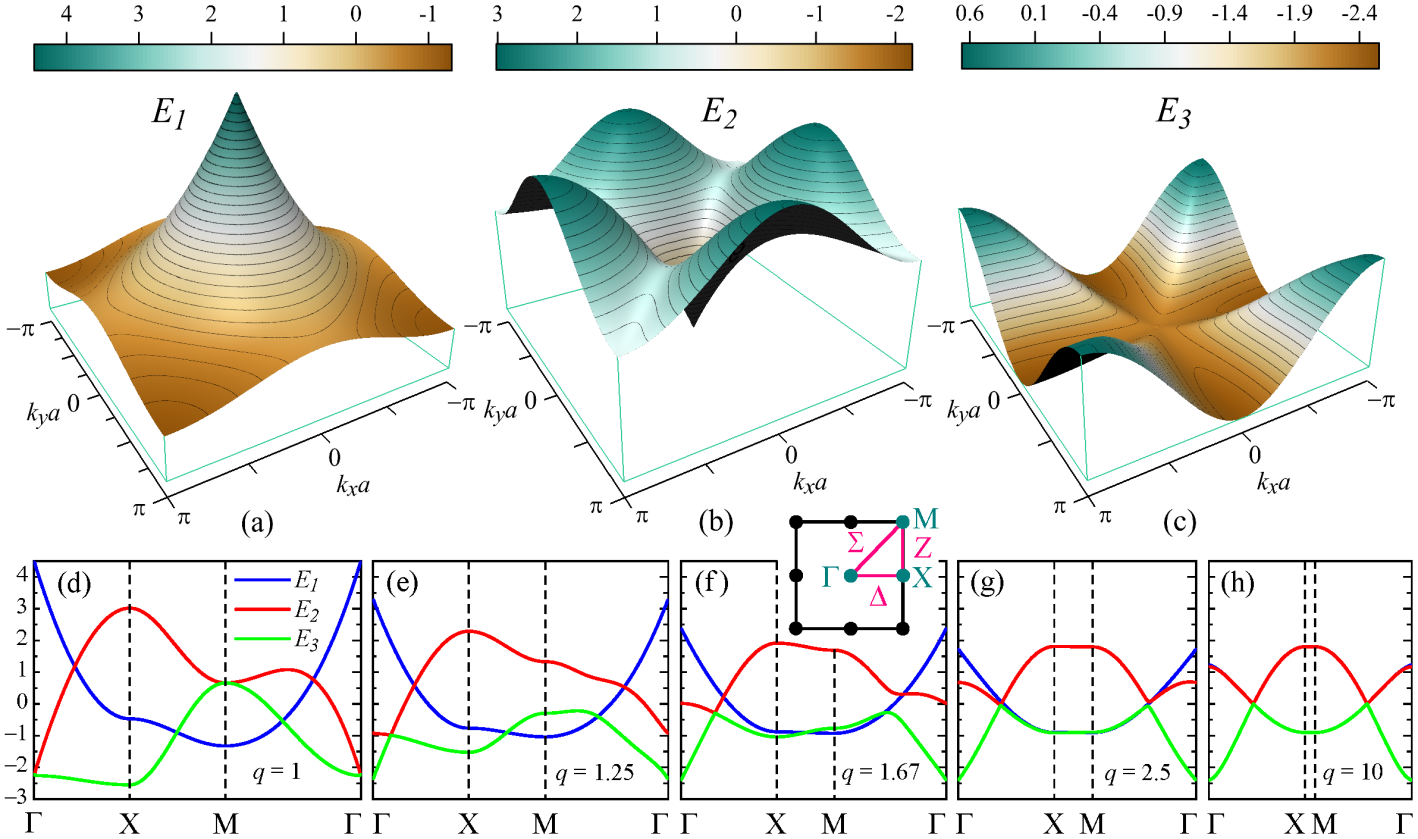
Exciton energy bands (a) *E*_1_, (b) *E*_2_, and (c) *E*_3_ in the first Brillouin zone of a two-dimensional quantum-dot supercrystal with square Bravais lattice [see Fig. 1(b)]. [(d)–(h)] Modifications of exciton bands upon transformation of (d) square lattice to [(e)–(h)] rectangular lattices with *q* = 1.25, 1.67, 2.5, and 10 [*q* = *b/a*, see Fig. 1(b)]. Blue, red, and green curves correspond to the first, second, and third bands, respectively. Inset in (f) shows symmetry points and symmetry lines in the reciprocal space.

The positions of the quantum dots in the square lattice in Fig. 1(b) are set by vectors 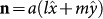, where *l* and *m* are integers and 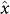 and 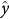 are the unit vectors. The corresponding exciton energy bands calculated from Eqs. (11) and (12) are shown in Figs. 2(a)–2(c). It is seen that the absolute maxima of the first, second, and third bands are located at points Γ, X, and M, respectively, whereas their absolute minima are at points M, Γ, and X. The second and third bands touch each other at points Γ and M, and cross the first band along the curves 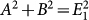 determining the states of a double degeneracy. The exciton energies at the symmetry points of the reciprocal space and the behavior of the energy bands along the symmetry lines Δ, Z, and Σ are summarized in Table 1. Unlike the first band, which has four simple saddle points at points X, the four simple saddle points of the second band lie on lines Σ due to the presence of local minima at points M. These four points are of particular practical interest, since they correspond to the infinite density of states, which diverges upon approaching their energy *E_c_* like −ln|*E* − *E_c_*|^71^. The third band exhibits only one, 3rd-order saddle point at the Brillouin zone center.

Distinctive features of the exciton energy spectrum in Fig. 2 are the sharp peak and sharp dip located at points Γ of the first and second bands. It can be readily seen from Eqs. (11) and (12) that ∇**_k_***E*_1_|**_k_**_ = **0**_ = ∇**_k_***E*_2_|**_k_**_ = **0**_ = **0**, which implies that the exciton group velocity at the peak and dip vanishes. These features are typical for the spectra of two-dimensional periodic systems and manifested, for example, as Dirac cones in the electronic spectrum of graphene^72^. The group velocity is also zero at the Brillouin zone boundary, where ∇**_k_***E_γ_* = **0** for all three energy bands^70^.

The band structure and the related properties of the supercrystal being considered may be engineered by varying either the size of the unit cell in the square lattice or the periodic arrangement of the quantum dots. In the first case, the topology of the exciton bands remains unchanged while their energies scale like 1/*a*^3^. The simplest way to modify the periodic arrangement of the quantum dots in the second case is to reduce the symmetry of the square lattice by stretching (or compressing) its unit cell along one of the primitive vectors; the resulting transformation is then described by a stretch (compression) factor *q* = *b*/*a*. Figures 2(d)–2(h) show how the exciton energy spectrum modifies when *q* is increased from 1 to 10. One can see that even a minor stretch *q* = 1.25 of the square lattice removes the degeneracy between the second and third bands at points Γ and M. At the same time, a new type of degeneracy appears in the vicinity of the Brillouin zone center. It is associated with the anticrossing of the second and third bands at the points where *A* = *B* = 0 and *E*_2_ = *E*_3_ = −*E*_1_/2.

As the stretch factor increases [see Figs. 2(e)–2(g)], the energy bands *E*_1_, *E*_2_, and *E*_3_ undergo a number of modifications. The least modification occurs to the first band, whose energy range simply shrinks while preserving the band's topology, whereas the changes in the shapes of the second and third bands are much more dramatic. The anticrossing region of bands 2 and 3 moves away from point Γ and starts manifesting itself along the Σ direction (which is clearly seen for *q* ≥ 1.67) while the energies of the degenerate states gradually approach zero. This significantly alters the topology of the two bands. In particular, point Γ becomes local maximum for the second band and absolute minimum for the third band. At the same time, points X become local minima for the third band, and 1st-order saddle points appear at points M of both energy bands.

For extremely large stretch factors (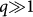), as in Fig. 2(h), the interaction between different rows of quantum dots becomes negligibly small and the two-dimensional supercrystal turns into a congregate of quantum-dot chains. It is easy to show that the exciton energies in the limit *q* → ∞ (*a* = const) are given by the expressions 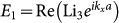 and *E*_2,3_ = (−*E*_1_ ± 3|*E*_1_|)/2, where Li*_n_x* is the common polylogarithm. The states of the first energy band are seen to be doubly degenerate throughout the first Brillouin zone (except for wave vectors corresponding to *E*_1_ = 0, for which they are triply degenerate): *E*_1_ = *E*_2_ = −*E*_3_/2 when *E*_1_ > 0, and *E*_1_ = *E*_3_ = −*E*_2_/2 when *E*_1_ < 0. Interestingly, the dependency *E*_1_(*k_x_*) is similar to the ordinary spectra of one-dimensional molecular crystals^63^, obtained using the approximation of nondegenerate intramolecular states.

If the square lattice undergoes a compression in the *y* direction, the dispersion of exciton energies increases due to the quantum dots' approachment. This feature provides additional flexibility in engineering the band structure of quantum-dot supercrystals. In the hypothetical limit of *q* → 0, we find that 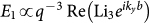.

### Supercrystals with hexagonal and centered rectangular lattices

**Figure 3:**
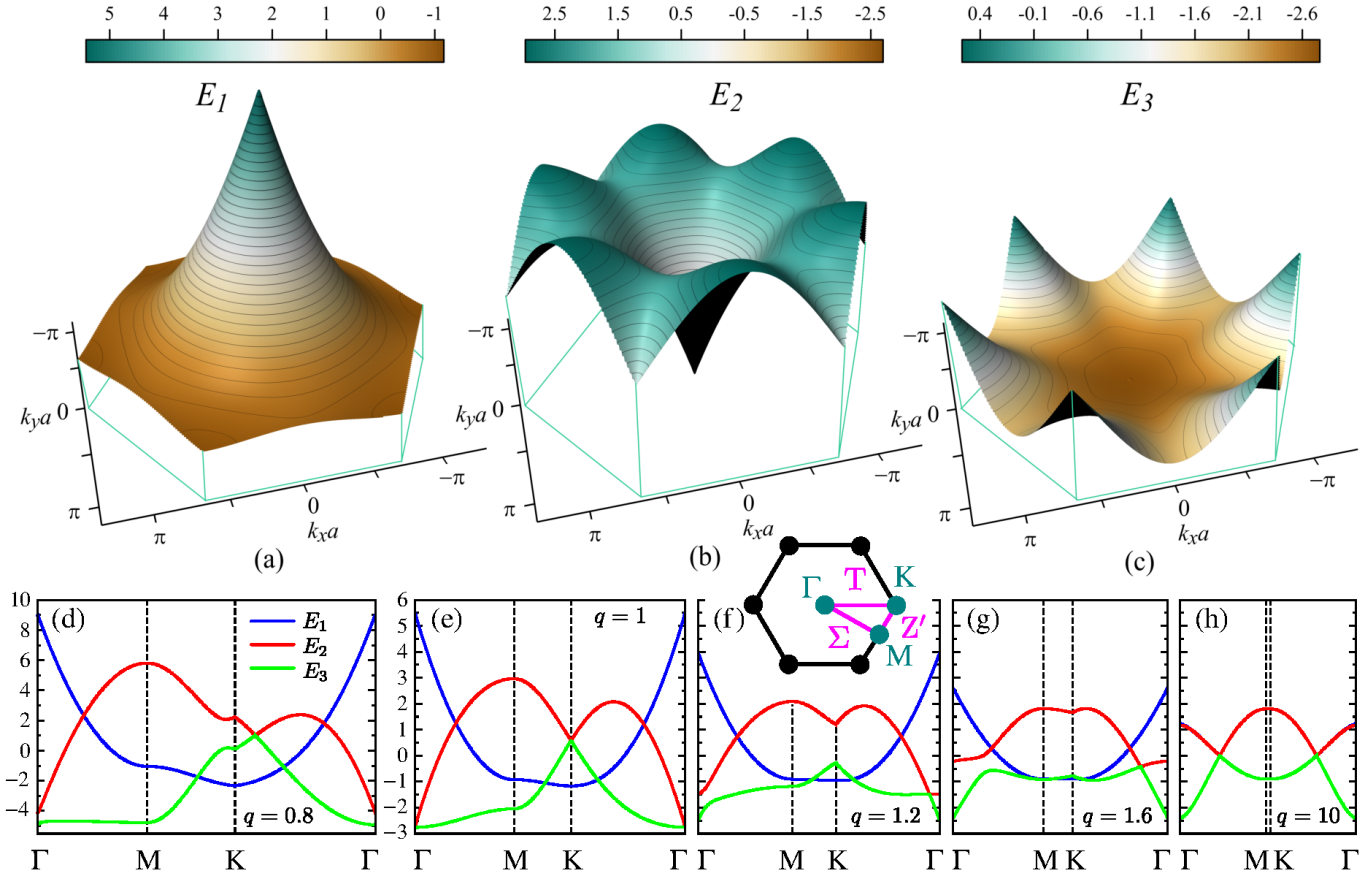
Exciton energy bands (a) *E*_1_, (b) *E*_2_, and (c) *E*_3_ in the first Brillouin zone of a two-dimensional quantum-dot supercrystal with hexagonal Bravais lattice [see Fig. 1(e)]. [(d)–(h)] Modifications of exciton bands upon transformation of (e) hexagonal lattice to [(d) and (f)–(h)] c-rectangular lattices with *q* = 0.8, 1.2, 1.6, and 10 [*q* = *b/a*, see Fig. 1(e)]. Blue, red, and green curves correspond to the first, second, and third bands, respectively. Inset in (f) shows symmetry points and symmetry lines in the reciprocal space.

We continue our analysis with the supercrystal of a hexagonal lattice shown in Fig. 1(e). The exciton bands of this supercrystal are plotted in Figs. 3(a)–3(c). Although the first Brillouin zone is now a regular hexagon, the minimal circuit formed by its symmetry points and symmetry lines is still a right triangle [see inset in Fig. 3(f)]. A comparison of the hexagonal energy bands with the energy bands in Fig. 2 reveals quite a few topological similarities. As can be seen from Table 2, the behavior of the first and second bands on triangle ΓMK fully coincides with that on triangle ΓXM of the square Brillouin zone, although the spread of energies in the former case is slightly larger due to the denser packing of quantum dots in the hexagonal lattice. The increase in the coordination number of a quantum dot alters the features of only the third energy band. Its points Γ and M turn an absolute minimum and the 1st-order saddle point, respectively, whereas the exciton's group velocity along the Σ direction becomes positive. In addition to that, six Dirac-like cones^72^,^73^ are formed at points K between the second and third bands. As it should be, the group velocity at the tips of the cones is zero, i.e., ∇**_k_***E*_2(3)_|_K_ = **0**.

Next, consider the situation in which changing **b** in the hexagonal lattice transforms it into the centered rectangular lattice shown in Fig. 1(f). In the special case of 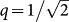, we obtain a square lattice considered earlier. When *q* is reduced beyond this value and approaches 1/2, the supercrystal splits into quantum-dot chains parallel to the *y* axis and its exciton energies diverge as 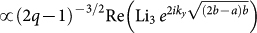. This divergency is seen to be slower than that for the square lattice due to the slower convergence of the quantum dots with the reduction of *q*.

If *q* is increased from 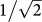 to unity, the exciton bands transform from the square type shown in Figs. 2(a)–2(d), through the intermediate stage in Fig. 3(d), to a hexagonal type shown in Fig. 3(e). This transformation removes the degeneracy from points Γ and K and results in the anticrossing of bands 2 and 3 along the T direction. The anticrossing point initially moves away from point K, but returns to it when *q* becomes sufficiently close to unity.

The modifications of the exciton energy bands with stretching of a hexagonal lattice is illustrated by Figs. 3(f)–3(h). Similar to the case of a rectangular lattice, points Γ and K become nondegenerate, the first energy band preserves its topology, and the anticrossings of bands 2 and 3 appear on lines T and Σ. For 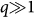, the exciton's dispersion becomes identical to that of the one-dimensional quantum-dot array [cf. Figs. 2(h) and 3(h)].

### Complex supercrystals

A complex supercrystal is a quantum-dot ensemble arranged in a lattice with a basis. If such a supercrystal contains *η* quantum dots in its unit cell, then it may be viewed as *η* supercrystals with the same simple lattice. Owing to the interaction between these supercrystals, the exciton spectrum of the entire nanostructure has *μ_c_* × *μ_v_* × *η* energy bands stemming from *μ_c_* × *μ_v_* exciton bands of the simple lattice through their splitting into *η* bands each. In the theory of molecular crystals, this type of energy-band splitting is known as *Davydov splitting*^63^.

**Figure 4:**
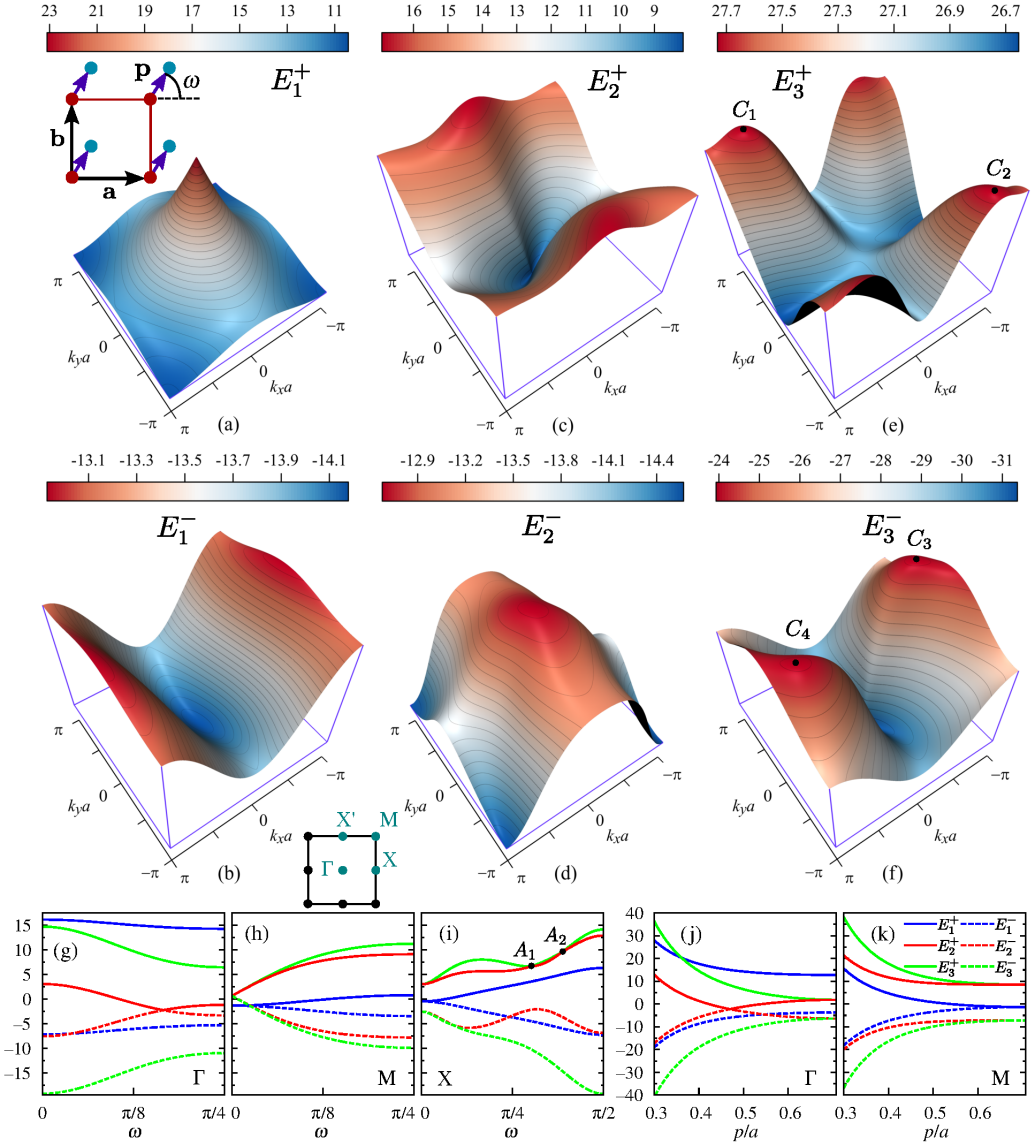
Exciton energy bands [(a) and (b)] 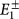, [(c) and (d)] 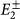, and [(e) and (f)] 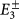 in the first Brillouin zone of a complex square lattice with two quantum dots in a unit cell for *p* = *a*/3 and *ω* = *π*/6, where *ω* is the angle between **a** and **p**, and *p* is the distance between quantum dots in a unit cell [see inset in (a)]. Symbols *C_μ_* (*μ* = 1,2,3,4) mark maxima at regular points of the reciprocal space. [(g)–(i)] Energies of six exciton bands at points Γ, M, and X as functions of *ω* for *p* = *a*/2. [(j) and (k)] Exciton band energies at points Γ and M *vs p* for *ω* = *π*/4. Blue, red, and green curves correspond to 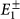, 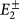, and 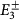, respectively. Inset in (b) shows symmetry points in the reciprocal space.

One of the easiest ways to create a complex supercrystal is to arrange pairs of quantum dots in a square lattice, as shown in the inset of Fig. 4(a). Assuming the positions of the two types of quantum dots in the supercrystal to be given by radius-vectors **r**_1_ = **n** and **r**_2_ = **n** + **p**, with **p** being a constant vector, we characterize the position of quantum dot 2 in the unit cell by its distance *p* = |**p**| from quantum dot 1 and the angle *ω* between vectors **p** and **a**. The six energy bands of the supercrystal with *p* = *a*/3 and *ω* = *π*/6 are plotted in Figs. 4(a)–4(f). It may be shown (see Methods) that the first two exciton bands, 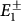, are due to the splitting of band *E*_1_ of the square lattice in Fig. 2(a). The shapes of bands 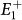 and *E*_1_ are seen to be similar, while the range of 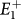 is almost twice that of *E*_1_, which is a result of the closer proximity of the quantum dots in the complex supercrystal. Since band 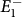 has much weaker dispersion than band 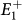, it is more sensitive to the relative positions of the quantum dots in the unit cell. Both bands exhibit absolute extrema at points Γ, absolute maxima at points X, and simple saddle points at points X′.

The other four bands, 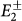 and 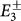, are the result of the splitting and intermixing of the second and third bands of the square lattice, which are shown in Figs. 2(b) and 2(c). The dispersion of bands 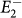 and 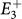 is seen to be much larger than that of bands 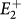 and 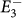, so that their shapes are strongly dependent on **p**. A distinctive feature of energy bands 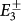 is extrema [*viz* maxima *C*_1_, *C*_2_, *C*_3_, and *C*_4_ in Figs. 4(e) and 4(f)] 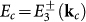 at regular points 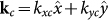 of the Brillouin zone. These extrema correspond to the *critical points*^71^,^74^ of the exciton energy spectrum, which is characterized by the density of states


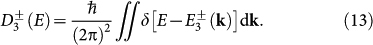
It is easy to show that 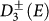 exhibits discontinuity steps at critical points and behaves at them as ∝ sgn(*E_c_* − *E*), where sgn(*x*) is the signum function. In addition to four maxima *C_μ_*, there are four critical points associated with the minima of bands 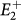 and 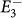. The critical points in the density of states play a crucial role in the interaction of excitons with each other and the external electromagnetic fields^75^,^76^.

The variation of exciton energies at points Γ, M, and X [shown in the inset of Fig. 4(b)] with angle *ω* is illustrated for *p* = *a*/2 by Figs. 4(g)–4(i). These dependencies may be extended to the entire range of *ω*, as well as to points X′, using the following relations obtained with symmetry considerations: 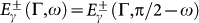, 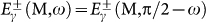, and 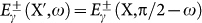. Several features peculiar to the exciton band structure are clearly seen from the figures. First, the exciton energies at points Γ and M are least prone to changes with *ω*. Second, the energies of the third and second bands at points Γ, M, and X satisfy the inequality 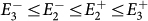 and, thus, may only touch each other at degenerate points like *A*_1_ and *A*_2_ in Fig. 4(i). Third, the energy gaps 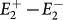 and 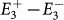 critically depend on *ω* at all symmetry points. These gaps decrease near *ω* = 0 due to the transformation of the complex supercrystal to an ordinary supercrystal with a simple rectangular lattice and *a* = 2*b*.

Figures 4(j) and 4(k) illustrate band transformations with the variation in distance between the quantum dots lying on the diagonal (*ω* = *π*/4) of the unit cell. As the figures suggest, the splittings of all bands decrease with distance and become zero for some of them when 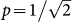. The latter case describes the situation of a simple supercrystal with a square lattice of spacing 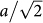. As before, the exciton energies formally diverge in the limit *p* → 0 due to the infinite convergence of the quantum dots in the supercrystal.

**Figure 5:**
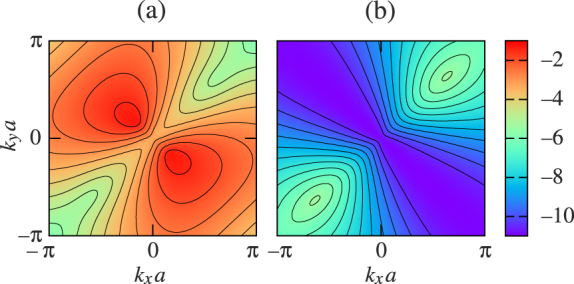
Critical points of exciton energy bands (a) 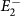 and (b) 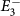 for a complex square lattice with two quantum dots in a unit cell [see inset in Fig. 4(a)]. It was assumed that *p* = *a*/2 and *ω* = *π*/4.

A unique feature of dispersion branches 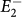 and 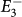 is that they may exhibit critical points at the tips of cone-like surfaces, which are similar to Dirac cones in Fig. 3 but located inside the first Brillouin zone. Figure 5 shows an example of such surfaces for a complex supercrystal with *p* = *a*/2 and *ω* = *π*/4. Each energy band is seen to exhibit two critical points along on of the diagonals of the first Brillouin zone. In real quantum-dot supercrystals such points would manifest themselves as sharp (but finite) peaks in the exciton density of states, and are therefore of primary importance for practical applications.

## Discussion

The above examples show how modification of the arrangement of the semiconductor quantum dots constituting a two-dimensional supercrystal can be used for engineering the spectra of its collective excitations, which ultimately affects the electromagnetic response of the supercrystal as a whole. In particular, we have demonstrated that a mere rearrangement of well-spaced quantum dots coupled through dipole–dipole interaction in a simple Bravais lattice offers broad control over the excitonic spectrum. Interestingly, the energy bands of excitons supported by the supercrystals with either square or hexagonal lattices were found to exhibit critical saddle points of the first order and the Dirac-like cones centered either at points Γ or the Brillouin zone boundaries. As the symmetry of these supercrystals is reduced, the energy spectrum undergoes drastic transformations that can be used for designing excitonic dispersion as desired for applications.

Additional degrees of freedom for controlling the supercrystal's properties can be introduced by assembling quantum dots into a periodic lattice with a basis. Our consideration of the square Bravais lattice with two quantum dots in a unit cell has revealed that the exciton energy spectrum features Davydov splitting, which is sensitive to the relative position of the quantum dots in the cell. A further complication of the structure *via* increasing the number of quantum dots in the unit cell—combined with the alterations in the symmetry of the lattice—provides almost unlimited degrees of freedom for engineering the physical properties of quantum-dot supercrystals.

Besides the flexibility in the supercrystal's design stemming from different arrangements of the quantum dots, there is a multitude of other ways to modify the electronic properties of the supercrystal and change its interaction with external electromagnetic fields. The variable parameters of a supercrystal include: (i) materials of the quantum dots; (ii) quantum dot shapes and dimensions; (iii) orientations of the quantum dots in space; (iv) positions and types of defects in the supercrystal lattice; (v) permittivity of the environment; and (vi) topology of the supercrystal. The above theory is readily modifiable to adequately describe the excitonic band structure in each of these situations.

For example, if the orientations of the quantum dots in a supercrystal are distributed in space according to function *g*(*ϑ, φ, ψ*), then the interaction operator in Eq. (8) should be averaged with respect to this distribution after being transformed by tensors 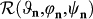 and 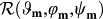 to crystallographic coordinates **R_n_** and **R_m_**. The averaging yields 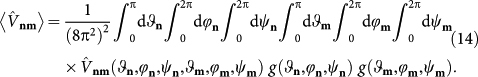
The solutions of the respective eigenvalue and eigenfunction problems are inextricably dependent on function *g*(*ϑ, φ, ψ*) and, hence, may be altered *via* the redistribution of the quantum dots' orientations.

Our theory enables one to readily estimate the typical energy dispersion of excitonic bands. Such an estimation requires knowing the scaling factor *C* in Eq. (10), which depends on the matrix element of a coordinate calculated on the electronic wave functions of the quantum dot's material. This matrix element is expressed through the Kane's parameter *P* of bulk semiconductor and the semiconductor's energy gap *E_g_* as 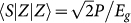
77. Specifically, for spherical InAs quantum dots (*ε*_QD_ = 12.25) located in the vacuum (*ε_h_* = 1), we have *P* = 1.8 eV × nm, *E_g_* = 354 meV (*T* = 300 K)^78^, and *Ca*^3^ ≈ 6.56 eV × nm^3^. For example, in the case of a square lattice with *a* = 20 nm, Table 1 shows that the energy bands in Fig. 2(a)–2(c) span over approximately 4.8, 4.3, and 2.6 meV, respectively. Since 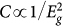, these values further increase (and may become comparable to the band gap) for quantum dots made of narrower band gap semiconductors. The possibility of tuning the exciton energy within large limits can benefit many types of photonic devices, including field-effect transistors^79^ and solar cells^62^.

The possibility of tuning the physical properties of quantum-dot supercrystals, and the enormous practical benefits associated with this ability, bring about the problem of finding the optimal structural and material parameters that will provide a desired configuration of the supercrystal's excitonic bands. Our work sets an important milestone in the theoretical studies aiming to resolve this important problem and, we believe, will eventually expand into a new research area of quantum-dot-based artificial materials with superior tuneable properties.

## Methods

Our calculation of the exciton energy bands of the complex lattice amounts to finding the eigenvalues of the Hermitian matrix


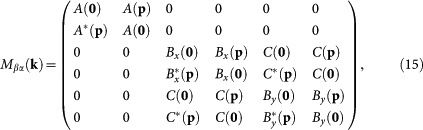
in which 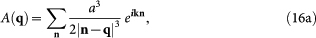

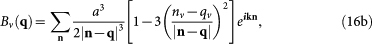
and


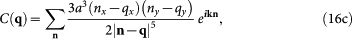
For **q** = **p** the summations in Eq. (16) are over all nodes of the lattice, while for **q** = **0** the node at the origin is to be excluded. Owing to a quasidiagonal block form of matrix *M_βα_* (**k**), the eigenvalues of each block can be found separately. The first two exciton bands from the 2 × 2 block are readily found to be 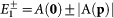, while the remaining eigenvalues are given by the Ferrari's formulas^80^.

Figures 2–5 were plotted using a commercial package MATLAB 2012a and assuming 200 × 200 nodes in the summations in Eqs. (11), (12), and (16).

## Author Contributions

I.D.R., A.V.B. and A.V.F. jointly suggested the study conducted by A.S.B. and V.K.T. Namely, A.S.B. and V.K.T. performed analytical calculations and analyzed the obtained expressions, drew the figures, and prepared the first draft of the manuscript. I.D.R., A.V.B. and A.V.F. supervised the study, contributed to the analysis and interpretation of the results, helped to formulate and present the research outcomes, and thoroughly edited the manuscript.

## Figures and Tables

**Table 1:** Behavior of exciton energy bands in the vicinity of symmetry points and along symmetry lines in the first Brillouin zone of a two-dimensional quantum-dot supercrystal with a square Bravais lattice. Numbers are the values of *E_γ_* at the corresponding points. The arrows 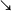, 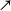, and 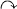 indicate a monotonous growth, monotonous decay, and growth followed by a decay, respectively

	Γ	X	M	Δ	Z	Σ
*E*_1_	absolute maximum 4.52	1st-order saddle point −0.47	absolute minimum −1.32	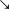	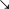	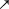
*E*_2_	absolute minimum −2.26	absolute maximum 3.02	local minimum 0.66	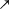	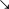	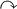
*E*_3_	3rd-order saddle point −2.26	absolute minimum −2.55	absolute maximum 0.66	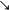	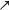	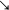

**Table 2:** Behavior of exciton energy bands around symmetry points and along symmetry lines in the first Brillouin zone of a two-dimensional quantum-dot supercrystal with a hexagonal Bravais lattice. All notations are the same as in Table 1

	Γ	M	K	Σ	Z′	T
*E*_1_	absolute maximum 5.52	1st-order saddle point −0.92	absolute minimum −1.17	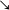	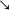	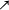
*E*_2_	absolute minimum −2.76	absolute maximum 2.97	local minimum 0.58	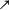	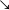	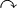
*E*_3_	absolute minimum −2.76	1st-order saddle point −2.05	absolute maximum 0.58	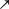	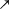	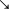
